# Antimicrobial Susceptibility and Fluoroquinolone Resistance Patterns of *Pseudomonas aeruginosa* Isolated from Canine Otitis Externa in Romania

**DOI:** 10.3390/antibiotics15020144

**Published:** 2026-02-02

**Authors:** Ionela Popa, Ionica Iancu, Vlad Iorgoni, Alexandru Gligor, Kalman Imre, Emil Tîrziu, Timea Bochiș, Călin Pop, Janos Degi, Andrei Ivan, Michael Dahma, Ana-Maria Plotuna, Gabriel Orghici, Viorel Herman, Ileana Nichita

**Affiliations:** 1 Department of Semiology, Faculty of Veterinary Medicine, University of Life Sciences “King Mihai I” from Timişoara, 300645 Timisoara, Romania; ionela.popa@usvt.ro (I.P.); timea.bochis@usvt.ro (T.B.); calinpop@usvt.ro (C.P.); 2 Department of Infectious Diseases and Preventive Medicine, Faculty of Veterinary Medicine, University of Life Sciences “King Mihai I” from Timişoara, 300645 Timisoara, Romania; vlad.iorgoni@usvt.ro (V.I.); alexandru.gligor@usvt.ro (A.G.); janosdegi@usvt.ro (J.D.); viorel.herman@fmvt.ro (V.H.); 3 Department of Food Safety and Hygiene, Faculty of Veterinary Medicine, University of Life Sciences “King Mihai I” from Timişoara, 300645 Timisoara, Romania; kalmanimre@usvt.ro; 4 Department of Microbiology, Faculty of Veterinary Medicine, University of Life Sciences “King Mihai I” from Timişoara, 300645 Timisoara, Romania; emiltirziu@usvt.ro (E.T.); andreialexandru.ivan.fmv@usvt.ro (A.I.); michael.dahma@usvt.ro (M.D.); ileananichita@usvt.ro (I.N.); 5 Department of Animal Nutrition, University of Life Sciences “King Mihai I” from Timişoara, 300645 Timisoara, Romania; anamaria.plotuna@usvt.ro; 6 Department of Veterinary Emergencies, Faculty of Veterinary Medicine, University of Life Sciences “King Mihai I” from Timişoara, 300645 Timisoara, Romania; gabriel.orghici@usvt.ro

**Keywords:** *Pseudomonas aeruginosa*, canine otitis externa, AMR, aminoglycosides, fluoroquinolones, zoonoses

## Abstract

**Background/Objectives:** Canine otitis externa (OE) is frequently complicated by *Pseudomonas aeruginosa* (*P. aeruginosa*) infections, which are often associated with treatment failure due to intrinsic and acquired antimicrobial resistance. This study aimed to assess the prevalence and antimicrobial susceptibility of *P. aeruginosa* isolates from dogs with OE in Timiș County, Romania, with a focus on aminoglycosides and fluoroquinolones, to provide region-specific, clinically relevant data and address potential public health implications. **Methods:** Exudate samples were collected from 435 dogs diagnosed with OE across multiple veterinary clinics between 2022 and 2025. *P. aeruginosa* isolates were identified using standard culture methods, and antimicrobial susceptibility was determined using the VITEK^®^ 2 Compact system according to CLSI VET01, Fifth Edition (2018) guidelines. Tested antibiotics included amikacin, gentamicin, enrofloxacin, marbofloxacin, and pradofloxacin. Resistance profiles were analyzed at both the individual antibiotic and class levels. **Results:** *P. aeruginosa* was isolated in 14.0% (61/435) of dogs. All isolates were susceptible to amikacin and gentamicin, whereas resistance to enrofloxacin and marbofloxacin was 27.9%, and pradofloxacin resistance reached 63.9%. A total of 24.6% of isolates were susceptible to all tested antibiotics. The most frequent multidrug-resistant combination among fluoroquinolones was ENR (R) + MAR (R) + PRA (R), observed in 23.0% of isolates. **Conclusions:** This study provides recent, region-specific data on *P. aeruginosa* prevalence and antimicrobial susceptibility in canine OE, offering clinically relevant insights into aminoglycoside and fluoroquinolone resistance. The findings highlight the potential public health significance of resistant *P. aeruginosa* strains at the human–animal interface and underscore the importance of antimicrobial stewardship in veterinary practice.

## 1. Introduction

Otitis externa (OE) is a common disease in dogs and represents a frequent reason for consultation in veterinary clinics worldwide. Secondary infections, notably those caused by *Pseudomonas* spp., most often *Pseudomonas aeruginosa* (*P. aeruginosa*), are key factors in disease progression, animal suffering, and treatment failure. Tissue changes, such as glandular hyperplasia and swelling, may be caused either by the primary factor or by secondary infections, further complicating disease management. In severe cases, medical treatments may be ineffective, necessitating surgical intervention, which can result in hearing loss and an increased risk of persistent infections [1]. *Pseudomonas* spp. are Gram-negative, saprophytic bacilli, highly resistant, occasionally encountered in cases of pyoderma and frequently in dogs with chronic otitis [2].

Infections with *P. aeruginosa* in the auditory canal in dogs are generally related to severe otitis externa. The clinical signs of otitis caused by *P. aeruginosa* are characterized by purulent discharge with marked erythema, erosions and ulcerations of the ear canal, frequently accompanied by bleeding, discomfort, and pain [3]. In chronic cases, rupture of the tympanic membrane is possible, as a result of the proteolytic enzymes produced by *P. aeruginosa* and of the lysozyme enzymes originating from inflammatory cells. These complications may lead to the occurrence of otitis media [3].

Due to intrinsic resistance mechanisms, *P. aeruginosa* exhibits natural resistance to various classes of antimicrobials. These mechanisms are represented by efflux pumps, low outer membrane permeability, and the production of a large amount of chromosomal AmpC β-lactamase [4,5]. More than that, *P. aeruginosa* is able to quickly develop resistance through loss of porin functionality, increase in the number of efflux pumps, production of a broad spectrum of β-lactamases, and modifications of antibiotic targets through mutations. Thus, *P. aeruginosa* can become resistant to any class of antimicrobials, using either a single mechanism or a combination of multiple mechanisms [5]. Biofilm can complicate the treatment of chronic infections [1,4,6].

The human–companion animal relationship is a major concern, as it raises a potential concern for the transmission of multidrug-resistant bacteria, and the global spread of these strains may pose a risk for both animal and human health [5]. Every year, drug-resistant *P. aeruginosa* triggers acute infections that take thousands of human lives across the world [7].

In the study conducted by Fernandes et al., it was reported that VIM-2 (Verona Integron-encoded Metallo-β-lactamase-2) producing *P. aeruginosa* was detected in three related sources: an infected dog, the dog’s owner, and several areas within their home. Genomic analyses showed that sequence type 233, a hospital-associated high-risk clone, was present across all samples, spanning the animal, human, and environmental niches. The findings point to household transmission of this VIM-2-positive strain following the patient’s discharge from the hospital, raising the possibility of a zooanthroponotic route [8].

Fluoroquinolones are a commonly used class of antibiotics for managing illnesses triggered by *P. aeruginosa.* Nevertheless, their therapeutic impact is declining as this pathogen rapidly develops resistance [9]. The growing prevalence of *P. aeruginosa* strains exhibiting resistance to antimicrobial agents has become a substantial therapeutic concern. Notably, fluoroquinolone resistance in this pathogen is frequently accompanied by broader multidrug resistance [10]. The risk of *P. aeruginosa* acquiring resistance to this antibiotic group is substantial, since fluoroquinolones are commonly chosen for therapy. As a result, the number of effective treatment options decreases, creating a widespread public health concern, particularly given the excessive use of antibiotics and the slow pace of discovering new clinically safe antimicrobial agents [11]. Resistance to amikacin and gentamicin is frequently observed, likely because these drugs are commonly used to manage otitis, as well as for corneal ulcers and pyoderma in dogs and cats. Careful administration of these antibiotics is necessary because of their potential ototoxic and nephrotoxic effects [12].

Antimicrobial resistance (AMR) has emerged globally as a critical challenge for public health, recognized as one of the foremost threats confronting modern healthcare systems [13,14,15,16,17]. In 2019, antimicrobial resistance was responsible for approximately 1.27 million deaths, and nearly five million additional fatalities were associated with infections that no longer responded to available drug treatments [14,18]. *P. aeruginosa* is recognized as one of the six major bacterial species that contribute substantially to mortality associated with antimicrobial resistance and is classified within the ESKAPE group [13]. The acronym “ESKAPE” refers to a group of clinically significant bacteria characterized by increasing virulence and multidrug resistance, including *Enterococcus faecium*, *Staphylococcus aureus*, *Klebsiella pneumoniae*, *Acinetobacter baumannii*, *Pseudomonas aeruginosa*, and species belonging to the *Enterobacter* genus. Collectively, these organisms account for a considerable proportion of infections acquired in healthcare settings and can survive antimicrobial treatment through diverse resistance mechanisms, ultimately leading to multidrug-resistant (MDR) phenotypes [19,20].

Antimicrobials commonly used in the management of canine otitis externa include topical aminoglycosides and fluoroquinolones, which represent the most frequently applied therapeutic options in routine clinical practice. These antimicrobial classes are particularly relevant for infections involving *P. aeruginosa*, while systemic therapy is generally reserved for selected severe or refractory cases. Accordingly, aminoglycosides and fluoroquinolones were selected for antimicrobial susceptibility testing in the present study. Canine otitis externa is commonly managed using topical antimicrobial agents, with aminoglycosides and fluoroquinolones representing the most frequently applied therapeutic classes in routine veterinary practice, particularly in infections involving *P. aeruginosa*. Systemic antimicrobial therapy is generally reserved for selected severe or refractory cases. Accordingly, aminoglycosides and fluoroquinolones were selected for antimicrobial susceptibility testing in the present study.

The aim of this study is to evaluate the prevalence and antimicrobial resistance profiles of *P. aeruginosa* isolated from cases of canine otitis externa, with a focus on resistance to aminoglycosides and fluoroquinolones. This study aims to provide data relevant for understanding local resistance patterns in veterinary practice and to contribute to the assessment of the zoonotic potential of resistant strains, highlighting their potential for transmission between animals and humans.

## 2. Results

### 2.1. Prevalence of P. aeruginosa

Out of a total of 435 dogs diagnosed with otitis externa, *P. aeruginosa* was isolated in 61 cases (14.0%).

Among the 435 dogs diagnosed with otitis externa, bacterial culture yielded either single or multiple bacterial species. The prevalence calculation (435 cases) included all dogs with otitis externa, regardless of whether infections were mono-microbial or polymicrobial.

Among the 61 cases positive for *P. aeruginosa*, infections were most frequently polymicrobial. Co-isolated organisms included *Staphylococcus pseudintermedius*, *Staphylococcus* spp., *Proteus* spp., and *Malassezia pachydermatis*. For prevalence and antimicrobial resistance analyses, *P. aeruginosa* was considered the target pathogen, and one isolate per dog was included, regardless of the presence of co-infecting organisms.

### 2.2. Antibiotic Resistance

Regarding antimicrobial resistance, the highest rate was observed for pradofloxacin (*n* = 39, 63.9%) (Table 1), while all isolates were susceptible to amikacin and gentamicin (*n* = 61, 0% resistance) (Table 1).

In total, 15 isolates (24.6%) were susceptible to all tested antibiotics (Table 2). Analysis of combined resistance profiles showed that the most frequent fully resistant combination among fluoroquinolones was ENR (R) + MAR (R) + PRA (R), observed in 14 isolates (23.0%) (Table 2).

Intermediate (I) isolates were interpreted according to CLSI guidelines. Clinically, an “I” result may indicate that the isolate could respond to higher doses or topical administration, but may be less reliably treated with standard systemic therapy. For the purposes of our study, intermediate isolates were considered susceptible in primary analyses. To aid clinical interpretation, we also provide a summary of susceptible versus non-susceptible isolates, combining intermediate and resistant results as non-susceptible (Table 3).

At the class level, an isolate was considered resistant to fluoroquinolones if it exhibited resistance to at least two antibiotics within the class; applying this criterion, 17 isolates (27.9%) were classified as resistant.

For transparency, class-level resistance was also calculated using the standard method, where resistance to any fluoroquinolone tested (enrofloxacin, marbofloxacin, or pradofloxacin) qualifies as resistance. Using this standard approach, 40 isolates (65.6%) were classified as fluoroquinolone-resistant.

Due to the limited number of antimicrobial classes tested, a formal classification of multidrug resistance was not applied in this study.

Given the unexpectedly high proportion of pradofloxacin-resistant isolates (63.9%) and the substantial percentage of isolates categorized as intermediate for enrofloxacin and marbofloxacin, these findings should be interpreted with caution. Antimicrobial susceptibility testing was performed using the VITEK^®^ 2 Compact system, which provides categorical interpretations based on embedded veterinary breakpoints rather than raw MIC values. In particular, for pradofloxacin, interpretation relied on manufacturer-embedded criteria, as internationally standardized CLSI breakpoints are currently unavailable. Consequently, breakpoint-related or method-dependent effects cannot be fully excluded. Future studies should include confirmation of selected fluoroquinolone resistance phenotypes using reference MIC-based methods, such as broth microdilution according to CLSI guidelines, to further validate these findings.

### 2.3. Year-by-Year Prevalence and Resistance Trends

The prevalence of *P. aeruginosa* in dogs with otitis externa varied slightly across the study period, with 11.1% (95% CI 5.5–19.8) in 2022, 12.0% (6.3–19.9) in 2023, 16.7% (10.5–25.0) in 2024, and 15.2% (9.5–22.9) in 2025 (Table 4). Although a minor increase in prevalence is observed in 2024, formal statistical testing for temporal trends was not performed.

Antimicrobial resistance patterns are summarized in Table 5. Aminoglycosides (amikacin and gentamicin) remained highly effective across all years. Resistance to fluoroquinolones was variable, with pradofloxacin resistance consistently high (58–74%) and a substantial proportion of isolates classified as intermediate for enrofloxacin and marbofloxacin (30–42% S, 35–42% I). For clinical interpretation, intermediate isolates were considered non-susceptible in secondary analyses. No obvious increase or decrease in resistance was apparent over the four-year period, although pradofloxacin resistance remains high and warrants continued monitoring.

### 2.4. Clinical Characteristics of P. aeruginosa-Positive Cases

Among the 61 *P. aeruginosa*-positive dogs, the majority had chronic or recurrent otitis externa (75.4%), with roughly half presenting unilaterally and half bilaterally. Underlying conditions included allergy (36.1%) and endocrinopathies (13.1%), while the remainder had other or unknown primary causes. Importantly, none of the dogs had received systemic or topical antibiotics in the two weeks prior to sampling, ensuring that the observed resistance patterns reflect natural susceptibility rather than recent treatment effects (Table 6).

## 3. Discussion

### 3.1. Prevalence in Comparison with Existing Literature

In this study, the prevalence of *P. aeruginosa* was 14.02%, which aligns closely with the findings of Park et al. [21], who reported a prevalence of 15.8%, as well as De Martino et al. [22], who observed 16%. Similar prevalence rates were reported by Petrov et al. [23] (17%) and Zamankhan Malayeri et al. [24] (10.87%). Conversely, higher prevalence rates were documented in other studies, including Bugden [25] (35.5%), Penna et al. [26] (31.6%) and Bıçakcıoğlu et al. [4] (26.7%), whereas Lyskova et al. [27] reported a lower rate of 7.2%. These variations may be attributed to differences in the canine populations studied, geographic regions, and clinical selection criteria applied [22,25,26].

### 3.2. Resistance to Aminoglycosides

At present, *P. aeruginosa* frequently exhibits resistance to aminoglycosides, and cases have been documented globally [28].

*P. aeruginosa* may develop aminoglycoside resistance via chromosomal changes as well as by acquiring resistance genes from other bacteria via horizontal gene transfer [29].

While resistance to aminoglycosides in *P. aeruginosa* can result from multiple factors, such as reduced cell permeability, ribosomal modifications or enhanced drug efflux, the primary mechanism involves enzymatic changes to the antibiotic’s active groups by aminoglycoside-modifying enzymes (AME) [28].

In the present study, *P. aeruginosa* exhibited no resistance to amikacin, with a resistance rate of 0%, consistent with findings reported by Zamankhan Malayeri et al. [24] and Petrov et al. [23], where resistance was also 0%. In contrast, Bıçakcıoğlu et al. [4] observed a slightly higher resistance rate of 9.3%, whereas Penna et al. [26] reported a substantially elevated rate of 70%, indicating significant variations among the studied populations. These discrepancies may be attributed to geographic differences, the antibiotic history of the populations examined, and methodological variations [23,24,26].

Topical use of aminoglycoside agents, including gentamicin, is commonly recommended for otitis externa cases involving Gram-negative microorganisms [30].

Regarding gentamicin resistance, *P. aeruginosa* isolates in the present study exhibited no resistance, with a rate of 0%, consistent with findings reported by Lyskova et al. [27], where resistance was likewise 0%. Similar low resistance rates have been documented in other studies, including 2% reported by Petrov et al. [23], 5% by Bugden [25], 8.7% by De Martino et al. [22], and 10% by Zamankhan Malayeri et al. [24]. In contrast, higher resistance rates were observed in other populations, with 20.93% reported by Bıçakcıoğlu et al. [4] and 43.3% by Mekić et al. [31]. These variations may reflect differences in local gentamicin usage patterns and the antibiotic history of the canine populations included in the respective studies.

### 3.3. Resistance to Fluoroquinolones

Fluoroquinolones are frequently administered empirically or selected as the initial treatment for various infections, such as ear infections, in companion animals, even though guidelines advise reserving them for cases where susceptibility testing shows that no first-line alternatives are available [32].

Bacteria can develop fluoroquinolone resistance through three main pathways: genetic mutations that modify drug target enzymes (topoisomerase IV and DNA gyrase), alterations that decrease membrane permeability, and the acquisition of plasmids carrying resistance genes [28].

In the present study, *P. aeruginosa* exhibited resistance to enrofloxacin in 27.9% of isolates. These results are comparable to those reported in other investigations, including 31% resistance observed by Rubin et al. [33] in *P. aeruginosa* isolates from dogs with otitis externa, otitis media, and pyoderma, 34.88% reported by Bıçakcıoğlu et al. [4] and 38% reported by Petrov et al. [23]. Conversely, higher resistance rates have been documented in other studies, such as 43.5% by De Martino et al. [22], 44.8% by Elfadadny et al. [34], 51.9% by Mekić et al. [31], and 71.4% by Lyskova et al. [27]. Furthermore, these findings differ from those reported by Zamankhan Malayeri et al. [24], where no resistance to enrofloxacin was observed in *P. aeruginosa* isolates (0%).

The prevalence of *P. aeruginosa* observed in the present study is broadly comparable to values reported in previous investigations of canine otitis externa, although variability among studies is evident. Similar susceptibility patterns, characterized by high activity of aminoglycosides and variable resistance to fluoroquinolones, have been described in canine-derived *P. aeruginosa* isolates, supporting the clinical relevance of the trends identified in the present study [35,36,37,38,39,40].

Regarding marbofloxacin resistance, in the present study, *P. aeruginosa* exhibited a resistance rate of 27.9%. These results are comparable to those reported by Rubin et al. [33], who observed a 27% resistance rate in *P. aeruginosa* isolates from dogs with otitis externa, otitis media, and pyoderma.

Differences in how fluoroquinolones remain effective have been observed across various regions in human populations, likely influenced by patterns of access to these drugs and their usage [2].

In addition to conventional antimicrobial resistance mechanisms, the limited susceptibility of *P. aeruginosa* to alternative or adjunctive antimicrobial approaches further illustrates the intrinsic therapeutic challenges associated with this pathogen [35,38,39,40].

Recent comparative in vitro studies have demonstrated differences in bactericidal activity among fluoroquinolones, indicating that susceptibility results should be interpreted within a pharmacodynamic context rather than assuming uniform activity across the class. In this respect, Blondeau and Fitch (2024) reported variable killing activity among fluoroquinolones, highlighting that resistance or reduced susceptibility to one agent may not uniformly reflect the activity of all compounds within the class [41].

### 3.4. Context of MDR P. aeruginosa and Implications of Observed Fluoroquinolone Resistance

Over the past few years, MDR *P. aeruginosa* strains have been increasingly reported, largely due to their ability to survive treatment with various antibiotics such as quinolones, aminoglycosides, and β-lactams. Additionally, the emergence of novel carbapenemase-producing strains has contributed to this growing concern [42]. In this study, only two classes of antibiotics, aminoglycosides and fluoroquinolones, were tested, limiting the ability to formally assess MDR. The observed resistance to fluoroquinolones represents a finding of interest, but broader conclusions regarding MDR patterns cannot be drawn from this dataset.

Although the present investigation focused exclusively on canine otitis externa, similar therapeutic challenges related to *P. aeruginosa* and antimicrobial resistance have been reported in otitis cases affecting other animal species. However, interspecies comparative analyses were beyond the scope of this study, which was intentionally designed to address canine clinical practice.

### 3.5. Strengths

This study provides recent, region-specific data on the prevalence and antimicrobial susceptibility of *P. aeruginosa* isolated from canine otitis externa in Timiș County, Romania, offering clinically relevant insights into resistance patterns to aminoglycosides and fluoroquinolones, antibiotics commonly used in veterinary practice. Moreover, the study adopts a One Health perspective by addressing antimicrobial resistance and highlighting the potential public health implications of resistant *P. aeruginosa* strains at the human–animal interface.

### 3.6. Limitations

The study was limited by the assessment of antimicrobial susceptibility to only two classes of antibiotics, preventing a comprehensive evaluation of multidrug resistance, and by its conduct in a single geographic region, which may restrict the generalizability of the findings to other areas. Additionally, the absence of molecular characterization precluded the identification of specific resistance mechanisms or transmission pathways, which limits conclusions regarding potential zoonotic spread.

Specifically, sequencing of QRDR regions (gyrA/parC) and screening for plasmid-mediated fluoroquinolone resistance and efflux/porin alterations were not performed; future studies could incorporate targeted sequencing of resistant isolates to provide mechanistic insights.

An additional limitation of this study is the lack of confirmatory minimum inhibitory concentration (MIC) testing using a reference method, such as broth microdilution. Antimicrobial susceptibility was assessed exclusively using an automated system (VITEK^®^ 2 Compact), which reports categorical results based on embedded interpretive criteria. This aspect is particularly relevant for fluoroquinolones, especially pradofloxacin, for which standardized CLSI veterinary breakpoints are not currently available. Therefore, the high resistance rate observed for pradofloxacin and the large proportion of intermediate results for enrofloxacin and marbofloxacin may partly reflect breakpoint-related or methodological effects. Validation of resistant isolates using reference MIC methods would strengthen the robustness of resistance estimates and should be considered in future investigations. It should be noted that susceptibility categories (S/I/R) for pradofloxacin were based on manufacturer-embedded criteria within the VITEK^®^ 2 system and may not be directly comparable to studies using other testing platforms or published MIC-based breakpoints.

Regarding case classification, as case classification was based on clinician judgment rather than strict temporal criteria, some misclassification between acute and chronic/recurrent cases is possible.

## 4. Materials and Methods

### 4.1. Study Design

Exudate samples from the external ear canal were obtained from dogs presenting otitis externa at multiple veterinary clinics across Timiș County, Romania, over the period 2022–2025.

### 4.2. Sample Collection

Samples were obtained from 435 dogs diagnosed with otitis externa, who exhibited clinical signs including malodor, erythema, pruritus, discharge, head shaking and localized pain. Cases of otitis externa were classified as acute or chronic/recurrent based primarily on clinician judgment. Chronic/recurrent cases typically included dogs with a history of repeated episodes or persistent signs despite prior treatment. No strict temporal cut-offs were applied due to the retrospective nature of the study.

Sterile swabs were used for sample collection, after which they were placed in Amies transport medium and maintained at 4 °C for no more than 24 h prior to laboratory processing. Although specimens were taken from both ears, only a single sample per animal was retained for analysis. When more than one *P. aeruginosa* strain exhibiting the same pattern of antimicrobial susceptibility was detected in samples from the same dog, only one isolate was included in the dataset. Additionally, there were instances in which *P. aeruginosa* was isolated from only one ear, while either no bacteria or a different species was recovered from the contralateral ear.

### 4.3. Species Isolation and Identification

The samples were inoculated onto selective and non-selective media (nutrient agar and cetrimide agar) and incubated at 35–37 °C for 18–24 h. Suspected *P. aeruginosa* colonies were chosen according to their distinctive morphological features, such as blue-green pigmentation, fruity odor, smooth or slightly undulated margins, and positive oxidase reaction. These presumptive colonies were subsequently subcultured onto fresh media until a pure culture was obtained, which was required for accurate identification and antimicrobial susceptibility testing.

Before conducting antimicrobial susceptibility assays, presumptive *P. aeruginosa* isolates were assigned a taxonomic identity through matrix-assisted laser desorption/ionization time-of-flight mass spectrometry (MALDI-TOF MS) on a Microflex™ instrument (Bruker Daltonik, Bremen, Germany). Protein extraction was carried out using a standard ethanol/formic acid procedure. Subsequently, 1 μL of the prepared protein solution was carefully placed on the surface of a MALDI target plate, and an equal volume of matrix solution, containing α-cyano-4-hydroxycinnamic acid at 10 mg/mL prepared in a solution of 50% acetonitrile combined with 2.5% trifluoroacetic acid, was added. The protein samples were analyzed with the Microflex™ mass spectrometer to generate the corresponding mass spectra, which were then processed through the MALDI BioTyper™ 3.0 analytical software. Identification of the isolates was performed by comparing the obtained spectra against the reference database provided by the manufacturer, applying the standard Bruker scoring criteria: scores ≥ 2.0 were considered reliable for species-level determination, while values between 1.7 and 2.0 indicated genus-level classification [43].

Before identifying the clinical samples, the spectrometer was calibrated and verified using the Bacterial Test Standard (BTS) together with a control strain (*Escherichia coli* ATCC 8739) following the procedures recommended by Bruker to ensure the accuracy of the acquired spectra. The bacterial species was then determined by comparing the protein spectra with the updated MALDI-TOF database, confirming *P. aeruginosa*.

### 4.4. Antimicrobial Susceptibility Testing (AST)

Antimicrobial susceptibility testing was performed with the VITEK^®^ 2 Compact system (bioMérieux, Marcy-l’Étoile, France), as recommended by the manufacturer. Prior to loading the samples, pure bacterial isolates were obtained, and the bacteria were suspended in sterile saline solution to prepare a uniform suspension, adjusting the turbidity to match the 0.5 McFarland standard. For the assessment of antimicrobial resistance, AST-P cards were utilized to generate the corresponding susceptibility profiles [44,45].

To determine antimicrobial susceptibility, isolates were tested against representatives of two antimicrobial classes: the aminoglycosides (amikacin and gentamicin) and the fluoroquinolones (enrofloxacin, marbofloxacin, and pradofloxacin).

Daily calibration and quality assurance of the VITEK^®^ 2 Compact system were carried out following the guidelines provided by the manufacturer, with *Pseudomonas aeruginosa* ATCC 27853 employed as the reference control strain. Interpretation of antimicrobial susceptibility results was performed based on the Clinical and Laboratory Standards Institute (CLSI) VET01, Fifth Edition (2018) criteria, which provide standardized criteria specifically designed for animal-derived bacterial isolates, ensuring reliability and accuracy of the testing process [44].

The antimicrobial classes included in this study were selected based on their frequent use in the clinical management of canine otitis externa and their relevance for antimicrobial stewardship in veterinary practice. Aminoglycosides, such as gentamicin, are commonly used topically, particularly in infections associated with this pathogen [22]. Fluoroquinolones are widely recognized as first-line agents for the treatment of canine ear infections, including both external and middle ear cases, when *P. aeruginosa* is involved [46,47,48]. Therefore, these antibiotics were chosen to reflect agents of high clinical relevance and to provide useful data for guiding appropriate therapy.

Antimicrobial susceptibility testing was performed using the VITEK^®^ 2 Compact system (bioMérieux, France) with veterinary AST-P cards. The system provides categorical susceptibility interpretations (susceptible, intermediate, resistant) based on embedded veterinary interpretive criteria rather than raw MIC values (Table 7).

Interpretation for amikacin, gentamicin, enrofloxacin, and marbofloxacin followed CLSI VET01, 5th edition (2018), as implemented by the VITEK^®^ 2 system. CLSI VET01 does not provide site-specific breakpoints for canine otitis externa; therefore, non-site-specific veterinary criteria were applied (Table 7)

Susceptibility interpretation for pradofloxacin was based on manufacturer-embedded veterinary criteria (bioMérieux), as internationally standardized CLSI breakpoints for this agent are not currently available (Table 7).

The VITEK^®^ 2 Compact system provides categorical antimicrobial susceptibility interpretations based on embedded veterinary criteria. CLSI VET01 does not provide site-specific breakpoints for canine otitis externa. Pradofloxacin susceptibility interpretation was based on manufacturer-embedded veterinary criteria, as internationally standardized CLSI breakpoints are not currently available for this agent.

### 4.5. Ethical Approval

Ethical approval for this research was obtained from the Bioethics Commission of the University of Life Sciences “King Mihai I” in Timișoara (Approval No. 611, dated 11 December 2025). This study did not involve any experimental procedures on live animals and adhered fully to all applicable ethical standards for animal research.

## 5. Conclusions

This study provides updated data on the prevalence and antimicrobial susceptibility patterns of *P. aeruginosa* isolated from canine otitis externa in Timiș County, Romania. *P. aeruginosa* was identified in 14.0% of examined cases, confirming its role as a significant opportunistic pathogen in chronic and severe forms of canine otitis externa.

Our results indicate that *P. aeruginosa* isolates from canine otitis externa in this study were susceptible to aminoglycosides, particularly in the context of topical therapy. These findings support the continued use of aminoglycosides in topical treatment for canine otitis externa, but caution is warranted as only two antibiotic classes were assessed and systemic efficacy cannot be inferred.

In contrast, a substantial proportion of isolates exhibited resistance to fluoroquinolones, particularly pradofloxacin, with more than one-quarter of isolates classified as fluoroquinolone-resistant at the class level.

Given the recognized zoonotic potential of *P. aeruginosa* and its classification within the ESKAPE group, these findings underscore the importance of routine culture and susceptibility testing in veterinary practice, as well as the need for prudent antimicrobial use to mitigate both therapeutic failure and the risk of transmission of resistant strains at the animal–human interface.

## Figures and Tables

**Table 1:** Antibiotic resistance of *P. aeruginosa* isolated from canine otitis externa.

Antibiotic	Total Isolates Tested	Susceptible	Intermediate (*n*/%)	Resistant (*n*/%)
Amikacin	61	*n* = 61/100%	*n* = 0/0%	*n* = 0/0%
Gentamicin	61	*n* = 58/95.1%	*n* = 3/4.9%	*n* = 0/0%
Enrofloxacin	61	*n* = 21/34.4%	*n* = 23/37.7%	*n* = 17/27.9%
Marbofloxacin	61	*n* = 21/34.4%	*n* = 23/37.7%	*n* = 17/27.9%
Pradofloxacin	61	*n* = 20/32.8%	*n* = 2/3.3%	*n* = 39/63.9%

Pradofloxacin interpretation based on manufacturer-embedded veterinary criteria (VITEK^®^ 2); standardized CLSI breakpoints are not currently available.

**Table 2:** Antibiotic resistance profiles of individual *P. aeruginosa* isolates.

Resistance Profile	Number of Isolates and Percentage
Susceptible to all antibiotics	15 (24.6%)
PRA (R)	6 (9.8%)
ENR (R) + MAR (R) + PRA (R)	14 (23.0%)
ENR (I) + MAR (I) + PRA (R)	16 (26.2%)
GEN (I) + ENR (R) + MAR (R) + PRA (R)	2 (3.3%)
ENR (I) + MAR (I)	4 (6.6%)
ENR (I) + MAR (I) + PRA (I)	2 (3.3%)
GEN (I) + ENR (I) + MAR (I) + PRA (R)	1 (1.6%)
ENR (R) + MAR (R) + PRA (S)	1(1.6%)

Legend: GEN—gentamicin; ENR—enrofloxacin; MAR—marbofloxacin; PRA—pradofloxacin; R = resistant, I = intermediate, S = susceptible.

**Table 3:** Fluoroquinolone susceptibility of *P. aeruginosa* isolates, considering intermediate (I) as non-susceptible.

Antibiotic	Susceptible (S)	Non-Susceptible (I + R)	% Non-Susceptible
Enrofloxacin	*n* = 21	*n* = 40	65.6%
Marbofloxacin	*n* = 21	*n* = 40	65.6%
Pradofloxacin	*n* = 21	*n* = 41	67.2%

**Table 4:** Annual prevalence of *P. aeruginosa* in dogs with otitis externa (2022–2025).

Year	Number of Dogs Examined	Number of Strains of *P. aeruginosa*	Prevalence (%)	95% CI
2022	90	10	11.1	5.5–19.8
2023	100	12	12.0	6.3–19.9
2024	120	20	16.7	10.5–25.0
2025	125	19	15.2	9.5–22.9
Total	435	61	14.0	10.9–17.5

**Table 5:** Annual resistance of *P. aeruginosa* (S/I/R) to tested antibiotics.

Antibiotic	Year	Susceptible (S) *n* (%)	Intermediate (I) *n* (%)	Resistant (R) *n* (%)
Amikacin	2022	10 (100%)	0 (0%)	0 (0%)
Amikacin	2023	12 (100%)	0 (0%)	0 (0%)
Amikacin	2024	20 (100%)	0 (0%)	0 (0%)
Amikacin	2025	19 (100%)	0 (0%)	0 (0%)
Gentamicin	2022	10 (100%)	0 (0%)	0 (0%)
Gentamicin	2023	12 (100%)	0 (0%)	0 (0%)
Gentamicin	2024	19 (95%)	1 (5%)	0 (0%)
Gentamicin	2025	19 (95%)	1 (5%)	0 (0%)
Enrofloxacin	2022	4 (40%)	4 (40%)	2 (20%)
Enrofloxacin	2023	5 (42%)	5 (42%)	2 (16%)
Enrofloxacin	2024	6 (30%)	7 (35%)	7 (35%)
Enrofloxacin	2025	6 (32%)	7 (37%)	6 (31%)
Marbofloxacin	2022	4 (40%)	4 (40%)	2 (20%)
Marbofloxacin	2023	5 (42%)	5 (42%)	2 (16%)
Marbofloxacin	2024	6 (30%)	7 (35%)	7 (35%)
Marbofloxacin	2025	6 (32%)	7 (37%)	6 (31%)
Pradofloxacin	2022	4 (40%)	0 (0%)	6 (60%)
Pradofloxacin	2023	5 (42%)	0 (0%)	7 (58%)
Pradofloxacin	2024	7 (35%)	1 (5%)	12 (60%)
Pradofloxacin	2025	4 (21%)	1 (5%)	14 (74%)

Pradofloxacin interpretation based on manufacturer-embedded veterinary criteria (VITEK^®^ 2); standardized CLSI breakpoints are not currently available.

**Table 6:** Clinical features of *P. aeruginosa*-positive otitis externa cases (*n* = 61).

Clinical Feature	Category	Number of Dogs (*n*)	Percentage (%)
Otitis type	Acute	15	24.6
	Chronic/recurrent	46	75.4
Laterality	Unilateral	32	52.5
	Bilateral	29	47.5
Primary underlying cause	Allergy	22	36.1
	Endocrinopathy	8	13.1
	Other/unknown	31	50.8
Prior antimicrobial exposure	None in past 2 weeks	61	100

**Table 7:** Antimicrobial susceptibility testing system and interpretive criteria applied in this study.

Antibiotic	Antimicrobial Class	AST System	AST Card Type	Interpretive Criteria Source	Site-Specific Breakpoints for Canine Otitis Externa	Interpretation Reported
Amikacin	Aminoglycoside	VITEK^®^ 2 Compact (bioMérieux)	AST-P (veterinary)	CLSI VET01, 5th ed. (2018), embedded in VITEK^®^ 2	No	Susceptible/Intermediate/Resistant
Gentamicin	Aminoglycoside	VITEK^®^ 2 Compact (bioMérieux)	AST-P (veterinary)	CLSI VET01, 5th ed. (2018), embedded in VITEK^®^ 2	No	Susceptible/Intermediate/Resistant
Enrofloxacin	Fluoroquinolone	VITEK^®^ 2 Compact (bioMérieux)	AST-P (veterinary)	CLSI VET01, 5th ed. (2018), embedded in VITEK^®^ 2	No	Susceptible/Intermediate/Resistant
Marbofloxacin	Fluoroquinolone	VITEK^®^ 2 Compact (bioMérieux)	AST-P (veterinary)	CLSI VET01, 5th ed. (2018), embedded in VITEK^®^ 2	No	Susceptible/Intermediate/Resistant
Pradofloxacin	Fluoroquinolone	VITEK^®^ 2 Compact (bioMérieux)	AST-P (veterinary)	Manufacturer-embedded veterinary criteria (bioMérieux)	No	Susceptible/Intermediate/Resistant

## Data Availability

The original contributions presented in this study are included in the article. Further inquiries can be directed to the corresponding author.

## Abbreviations

The following abbreviations are used in this manuscript:

*P. aeruginosa*	*Pseudomonas aeruginosa*
OE	Otitis externa
AMR	Antimicrobial resistance
MDR	Multidrug-resistant
AME	Aminoglycoside-modifying enzymes
MALDI-TOF MS	Matrix-assisted laser desorption/ionization time-of-flight mass spectrometry
BTS	Bacterial Test Standard
AST	Antimicrobial Susceptibility Testing
CLSI	Clinical and Laboratory Standards Institute
